# Atopic, seborrheic, and contact dermatitis burden in G20 countries, 1990-2023, with projections to 2050

**DOI:** 10.1016/j.jdin.2026.06.016

**Published:** 2026-07-01

**Authors:** Yilin Wang, Zhongzhi Wang

**Affiliations:** Department of Dermatology, Shanghai Fourth People's Hospital, School of Medicine, Tongji University, Shanghai, China

**Keywords:** atopic dermatitis, contact dermatitis, epidemiology, G20, Global Burden of Disease, prevalence, projection, seborrheic dermatitis

*To the Editor:* Inflammatory dermatoses, such as atopic dermatitis, seborrheic dermatitis (SD), and contact dermatitis (CD), are among the most prevalent chronic skin diseases globally, significantly contributing to the burden of nonfatal diseases.^1^^,^^2^ These conditions are characterized by chronic inflammation, recurrent flares, and substantial impairment in quality of life, primarily due to pruritus, sleep disturbances, and psychosocial stress. Despite their high prevalence, comparative epidemiological assessments across major global economies remain limited. The Group of Twenty (G20) countries, which encompass a significant portion of the global population and health expenditure, are crucial for understanding global disease patterns. Utilizing data from the Global Burden of Disease Study 2023, we evaluated the burden of atopic dermatitis, SD, and CD across G20 countries from 1990 to 2023 and projected trends up to 2050.

Data on incidence, prevalence, disability-adjusted life years and years lived with disability were obtained from the Global Burden of Disease Study 2023 database.^1^ Age-standardized rates were used for comparisons across countries, sexes, and age groups. Temporal trends from 1990 to 2023 were analyzed, and projections through 2050 were generated using autoregressive integrated moving average time-series models. Subgroup analyses were conducted by sex and age. Temporal trends from 1990 to 2023 were analyzed, and projections of prevalence through 2050 were generated using time-series modeling. Subgroup analyses further examined variations by sex and age to better characterize high-risk populations.

In 2023, inflammatory dermatoses remained highly prevalent in G20 countries. Atopic dermatitis affected approximately 180.2 million individuals and was associated with 7.85 million disability-adjusted life years.^3^ SD affected 20.9 million individuals, while CD accounted for 122.0 million cases and 2.98 million disability-adjusted life years.^4^^,^^5^ Country-specific estimates are detailed in the Supplementary Tables, available via Mendeley at https://data.mendeley.com/drafts/pdjb8x53gn. Substantial geographic heterogeneity was observed (Fig 1). Russia exhibited the highest burden of SD burden, whereas China contributed the largest share of CD cases among G20 countries. Females consistently showed higher incidence and prevalence than males across most regions. Disease burden was concentrated in younger populations, particularly those aged 0-49 years, indicating a long-term impact across the lifespan (Fig 1).

**Fig 1:**
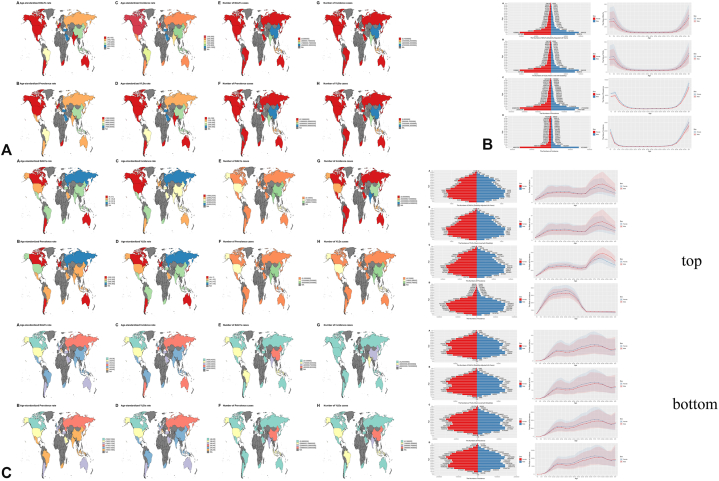
Burden of inflammatory dermatoses in G20 countries in 2023. **A,** Geographic distribution of DALYs, incidence, prevalence, and YLDs for AD. **B,** Age and sex-specific distribution of these measures for AD. **C,** Corresponding analyses for SD (*top*) and CD (*bottom*). *AD*, Atopic dermatitis; *CD*, contact dermatitis; *DALYs*, disability-adjusted life years; *G20*, Group of Twenty; *SD*, seborrheic dermatitis; *YLDs*, years lived with disability.

From 1990 to 2023, age-standardized incidence and prevalence rates have remained relatively stable across most G20 countries. However, the absolute number of cases increased markedly and may be associated with population growth and demographic changes.^1^ Projections to 2050 suggest that although age-standardized rates may remain stable, the total burden will persist at high levels, with continued increases in prevalence in several countries (Fig 2).

**Fig 2:**
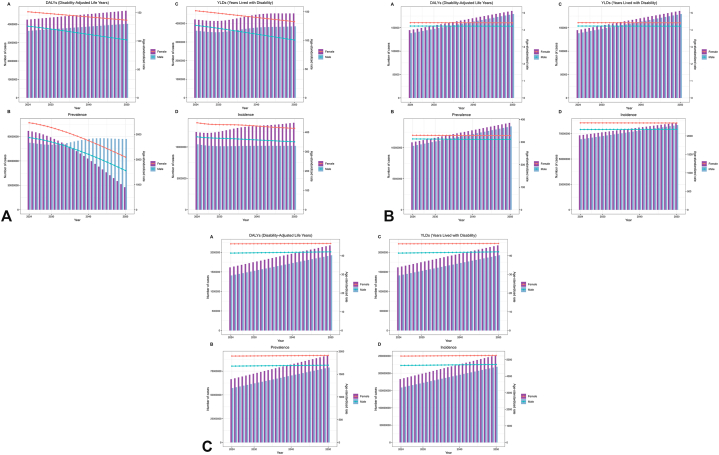
Projected trends in inflammatory dermatoses in G20 countries through 2050. **A,** Forecasted DALYs, incidence, prevalence, and YLDs for AD. **B,** Corresponding projections for SD. **C,** Corresponding projections for CD. *AD*, Atopic dermatitis; *CD*, contact dermatitis; *DALYs*, disability-adjusted life years; *G20*, Group of Twenty; *SD*, seborrheic dermatitis; *YLDs*, years lived with disability.

The persistent burden of inflammatory dermatoses underscores the necessity for improved prevention and management strategies. Given their chronic and relapsing nature, these conditions remain important contributors to nonfatal disease burden in G20 countries. Further studies are needed to evaluate effective prevention and management strategies, particularly among high-risk populations.

## Conflicts of interest

None disclosed.
